# Hydrogen sulfide generated by cystathionine gamma lyase inhibits lysyl oxidase and protects against calcific tendinopathy

**DOI:** 10.1016/j.jot.2026.101082

**Published:** 2026-03-25

**Authors:** Elodie Faure, Ilaria Bernabei, Driss Ehirchiou, Philipp Michel, Daniel Kronenberg, Thomas Pap, Giuseppe Cirino, Richard Stange, Nathalie Busso, Sonia Nasi

**Affiliations:** a Service of Rheumatology, Department of Musculoskeletal Medicine, Lausanne University Hospital, Switzerland; b Trauma, Hand and Reconstructive Surgery, University Hospital Münster, Germany; c Department of Regenerative Musculoskeletal Medicine, Institute for Musculoskeletal Medicine, University Hospital Münster, Germany; d Department of Molecular Medicine, Institute for Musculoskeletal Medicine, University of Münster, Germany; e Department of Pharmacy, University of Naples Federico II, Naples, Italy; f Department of Rheumatology and Immunology, Semmelweis University Budapest, Budapest, Hungary

**Keywords:** Cystathionine-gamma-lyase, Hydrogen sulfide, Lysyl oxidase, Lysyl oxidase inhibition, Pathologic calcification, Tendinopathy

## Abstract

**Background/objective:**

Increased lysyl oxidase (LOX) activity favors pathologic cartilage and vessel calcification. LOX promotes disease through enhanced collagen cross-linking, inflammation, reactive oxygen species (ROS) production, cell trans-differentiation, and fibrosis. This study investigates the therapeutic potential of cystathionine gamma lyase (CSE)-generated hydrogen sulfide (H_2_S) to inhibit tendon calcification by targeting LOX in human samples and murine models of calcific tendinopathy (CT).

**Methods:**

Human shoulder supraspinatus tendons with varying degrees of CT were analyzed using Alizarin Red staining and LOX and CSE immunohistochemistry to evaluate the correlation between CSE and LOX/calcification. Mechanistic studies were performed using wild-type (WT) and CSE knockout murine tenocytes cultured in calcification-inducing medium with or without H_2_S donors or the LOX inhibitor β-aminopropionitrile (BAPN). Achilles tendon CT was induced in WT and CSE knockout mice via surgical intervention or aging. Tendon calcification, LOX expression, biomechanical integrity, and transcriptomic changes were assessed. Persulfidation of total proteins and recombinant human LOX (rhLOX) was measured using the dimedone-switch method.

**Results:**

An inverse correlation between CSE levels and LOX/calcification was observed in human tendons and in the surgery-induced CT murine model. In murine tenocytes and in the aging murine model, CSE deficiency led to increased LOX expression, enhanced calcification, and reduced tendon biomechanical integrity.Transcriptomic analysis confirmed the negative association between CSE and LOX in murine CT. Mechanistically, H_2_S increased total cellular protein persulfidation, including rhLOX, resulting in inhibition of its enzymatic activity.

**Conclusion:**

Dysregulated LOX activity is a key driver of calcific tendinopathy. CSE-generated H_2_S effectively suppresses LOX activity, highlighting its potential as a therapeutic strategy for CT and other calcification-related disorders.

**The translational potential of this article:**

This study identifies LOX as a therapeutic target in CT and supports H_2_S as a promising treatment strategy for this condition.

## Graphical abstract

**Proposed mechanistic model** In healthy tendon, CSE generates enough H_2_S to inhibit LOX-induced calcification, depicted in red. Contrarily, in tendon with trauma or in aged tendons, CSE-generated H_2_S is diminished ultimately leading to increased LOX activity and calcification. Overall, H_2_S could be of therapeutic relevance in calcific tendinopathy.

**Figure:**
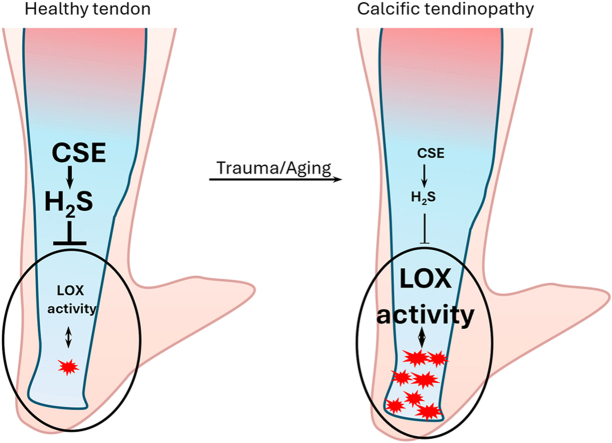

## 1 Introduction

H_2_S is a gasotransmitter endogenously produced by mammalian cells, able to diffuse through cell membranes and elicit biological functions [1]. H_2_S is endogenously synthesized by three enzymes: cystathionine beta synthase (CBS), cystathionine gamma lyase (CSE) and 3-mercaptosulfur transferase (3-MST) [1]. Alternatively, H_2_S can be exogenously administered in the form of H_2_S donors (sodium hydrogen sulfide (NaHS), or synthetic compounds (GYY4137), or sodium thiosulfate (STS)) [1]. One of the main mechanism for H_2_S signalling is the post-translational modification of protein cysteine residues (P-SH), called S-sulfhydration (or persulfidation, P-SSH) [2]. This can result in gain or loss of function of the persulfidated protein. For example, persulfidation of GAPDH at its ^150^Cys leads to higher enzymatic activity [3].

Among the diverse roles of H_2_S, its anti-calcification effect is notable. This protective effect is attributed to H_2_S's ability to block pro-calcification mechanisms within cells, including inflammation [[4], [5], [6]] and reactive oxygen species (ROS) production [[5], [6], [7]]. We showed that the H_2_S donor STS inhibits cartilage calcification in murine osteoarthritis, likely via decreasing interleukin-6 (IL-6) and mitochondrial ROS production by chondrocytes [5]. Likewise, 3-MST or CSE deficient murine chondrocytes calcify more and produce more IL-6 and ROS than WT (wild-type) chondrocytes [6,8]. Moreover, H_2_S blocks trans-differentiation of non-calcifying vascular smooth muscle cells into calcifying cells [9]. In contrast, Yuan et al. reported that exogenous H_2_S accelerates trauma-induced heterotopic ossification in tendons [10]. However, their study employed a relatively high NaHS dose (5 mg/kg daily for 14 days), evaluated endogenous H_2_S solely via propargylglycine (a relatively non-selective CSE inhibitor), and used murine and human samples not directly comparable to ours [10]. Therefore, the physiopathological role of endogenous CSE-derived H_2_S in calcific tendinopathy is still unclear.

Lysyl oxidases (LOX(L)) are a family of copper-dependant enzymes composed of lysyl oxidase (LOX) itself and four lysyl oxidase-like proteins (LOXL1, -2, -3, -4) [11]. LOX(L) share a conserved C-terminal catalytic domain and a variable N-terminal domain. Importantly, LOX(L) contain disulfide bridges in their active site, which are fundamental for their tertiary structure and catalytic activity [12]. Proper LOX(L) activity is essential for collagen cross-linking, which maintains extracellular matrix (ECM) homeostasis by stabilizing collagen and elastin fibrils [[13], [14], [15]]. Interestingly, LOX(L) enzymes can also enter in the cells to exert intracellular functions. For instance, LOX and LOXL2 can translocate into nuclei to modify histones and act as transcriptional regulators [16]. In scleroderma, cytosolic LOX induces inflammation by upregulating the expression of the c-Fos transcription factor and promoting its nuclear translocation, subsequently inducing interleukin 6 (*IL-6*) expression [17]. LOX has been also associated with intracellular ROS generation as arteries from TgLOX mice exhibited increased vascular H_2_O_2_ levels while BAPN, a pan-inhibitor of LOX(L), prevented oxidative stress in hypertensive models [18]. We and others recently demonstrated that increased LOX(L) activity favours pathologic calcification in cartilage [19] and in vessels [20,21]. This could be explained by LOX(L) mediated increase in collagen cross-links, inflammation, ROS production, cell trans-differentiation and fibrosis. In tendons, LOX-mediated cross-linking is essential during physiologic development to ensure proper collagen fibril maturation, linear fiber growth, and optimal material properties [22]. Aberrant LOX-dependent cross-linking, leading to irregular collagen fibril morphology, is associated with severe congenital connective tissue disorders affecting tendons, including Ehlers–Danlos syndrome, osteogenesis imperfecta, and Kuskokwim syndrome [22]. Age-related alterations in LOX-mediated cross-linking have also been reported in individuals over 65 years of age and are associated with impaired tendon material properties and increased susceptibility to injury [22]. In tendinopathies, LOX has been identified as a strong candidate risk gene; however, its specific role in tendon calcification remains largely unexplored [23]. To date, no studies have specifically investigated the role of LOX in tendon calcification. Therefore, determining whether LOX exerts a deleterious effect in this context, and consequently whether LOX(L) inhibition may represent a therapeutic strategy for calcific tendinopathy, is of considerable interest. To date, three classes of LOX(L) inhibitors exist: small molecule inhibitors [24], monoclonal antibodies, and copper chelators [11]. Nevertheless, most of them do not target a specific LOX(L) or are still in pre-clinical or early clinical phases. Moreover, they have only been assessed in cancer or fibrotic diseases [11].

In this study, we first identified a negative association between the H_2_S-generating enzyme CSE and LOX/calcification in human samples of calcific tendinopathy. To further investigate this relationship, we employed both *in vitro* and *in vivo* models of the disease. Specifically, we utilized CSE KO mice to elucidate the mechanistic role of endogenous CSE-derived H_2_S. We hypothesized that the observed inverse correlation may be mediated by H_2_S-dependent inhibition of LOX activity.

## 2 Methods

### 2.1 Experimental design

The objectives of this study were: i) to establish the association between CSE/H_2_S levels and LOX/calcification levels in human calcific tendinopathy; ii) to evaluate whether H_2_S anti-calcifying effects could be explained by its ability to inhibit LOX activity. Starting from the human *ex vivo* evidence, we used a reverse translational approach based on *in vitro* and *in vivo* murine models, since it is not ethically possible to perform these studies in human. All studies involving human samples and animals were approved by the appropriate ethics review boards. Informed consent was obtained from all participants before the operative procedure. A key limitation affecting the generalizability of this study is the absence of sex- or gender-specific analyses, as the cohort was not stratified by sex. Future studies should address sex stratification, which will likely require a larger number of human samples, potentially achievable through multi-center collaborations to optimize tissue collection. Indeed, another limitation is the relatively small human sample size (N = 16). Nevertheless, this represents a comparatively substantial cohort in the context of human tendon research. The collection of human tendon tissue is inherently challenging, as tendons are rarely excised during surgical procedures due to the predominantly conservative approach to tendon management in clinical practice. For *in vivo* experiments, sample size was estimated through power analysis, considering previous studies in similar experimental conditions, where we expected a 50% difference in tendon calcific deposits. Considering our experimental standard deviation, analysis by post-hoc one-way ANOVA followed by bilateral one-sample t-test, an α-error of 0.05 and a power of ≥0.8, n = 7 mice/group was the good sample size to adopt. The mice were randomly assigned to experimental groups and divided into two cages with three and four mice each. The allocation, data acquisition, and analysis were conducted by two independent investigators in a blinded manner. Exclusion criteria prior to completing the experiment included significant discomfort or adverse effects observed in the animals. Trained personnel monitored the animals daily and assessed their health status based on veterinary guidelines, and none of the animals met the exclusion criteria. For some immunohistochemical staining, some mice were not analyzed due to technical issue in the processing of the sample. *In vitro*, media were changed every three days. In RT-PCR experiments, we excluded genes with a Ct value over 38. For each experiment, the sample size reflected the number of independent biological replicates and is indicated in the figure legend.

### 2.2 Murine tenocytes isolation

Achilles tendons of 6-week-old mice were dissected from hind limbs. Bone and muscle were carefully removed prior to a two-step digestion in Collagenase IV (Witec AG) in 3 mg/mL αMEM Eagle (PAN biotech)+2 mM glutamine+1% penicillin-streptomycin (P/S). After centrifugation, supernatant was removed and pellet suspended and replated in a 25 cm^2^ flask, in αMEM Eagle+ 2 mM glutamine+10% FBS+50 μg/ml L-ascorbic acid- 2-phosphate (Sigma–Aldrich)+1% P/S. Adherent tenocytes were amplified and used at P2. Medium was changed every 3 days of culture. The expression of Scleraxis, Tenomodulin, Collagen type I, and Collagen type III in tenocytes was assessed by qRT-PCR and compared to their expression in NIH3T3 fibroblasts, confirming the effective differentiation of these cells into tenocytes (Fig. S1A).

### 2.3 H_2_S detection

H_2_S production in WT and CSE KO tenocytes was detected by the lead sulfide method as previously described [25]. Briefly, tenocytes (2∗10^5^ cells/cm^2^) were cultured in a 96 well-plate covered with the lead acetate paper for 24 h at 37 °C. The brown color generated on the paper corresponded to H_2_S produced by cells.

### 2.4 Calcification experiments

Tenocytes were plated at 2∗10^5^ cells/cm^2^ and cultured in different calcifying media (CM). For short time experiments (24 h), CM consisted of αMEM Eagle (PAN-Biotech)+2 mM glutamine+10% FBS+50 μg/ml L-ascorbic acid 2-phosphate (Sigma–Aldrich) + secondary calciprotein particles (CPP2, final concentration equivalent to 100 mg/mL calcium [26])). For long time experiments (14 days), CM consisted of BGJb GlutaMAX medium (Gibco)+10% FBS, 50 μg/ml L-ascorbic acid 2-phosphate (Sigma–Aldrich)+20 mM β-glycerol phosphate (Sigma–Aldrich). The choice of calcification media and experimental duration was based on a scientifically driven rationale. H_2_S experiments had to be short-term due to gas volatility, so CPP2, highly reactive precursors of calcification are the ideal method to induce calcification within 24 h. In contrast, LOX activity or inhibition studies required longer culture periods to allow matrix production and cross-linking, making β-glycerophosphate supplementation the preferred method to induce calcification over approximately 14 days. Where indicated, cells were treated with: 25 mM Sodium Thiosulfate (STS), 100 μM NaHS, 100 μM GYY4137 or 500 μM β-aminoproprionitrile (BAPN) (all Sigma–Aldrich). At the end of the experiments, supernatants were removed and assayed for cytotoxicity (LDH) and ELISA. Cell monolayers were washed in PBS, fixed in 10% formol and stained with Alizarin red for quantification of calcification [27]. Alternatively, monolayers were washed in PBS and dissolved in TRIzol (500μl/10^6^) for RNA extraction or lysed in RIPA buffer for western blotting.

## 3 ELISA

Collected supernatants were assayed for murine IL-6 secretion (eBioscience), following manufacturer's instructions.

### 3.1 Western blotting

WT and CSE KO tenocytes were plated at 2.5∗10^5^ cells/cm^2^ or 0.5∗10^5^ cells/cm^2^ and cultured for 24 h or 14 days in CM, respectively. Tenocyte lysates were separated using SDS-PAGE (mPAGE 4-20% Bis-Tris gels, Millipore). Proteins were transferred into immobilon P-PVDF membranes using a wet blotting technique. Pro- and mature-LOX were detected using the LOX antibody (17958-1-AP, Proteintech) followed by incubation with anti-rabbit IgG HRP conjugate secondary antibody (W401B, Promega). β-actin mouse anti-mouse antibody (8H10D10, Cell signaling) and mouse anti-mouse IgG HRP conjugate secondary antibody were used (W402B, Promega) to detect the housekeeping protein. Bands were revealed with ECL (Immobilon Western Chemiluminescent HRP Substrate, Millipore) and detected with Fusion FX.

### 3.2 RNA extraction, cDNA synthesis and qRT-PCR analysis

RNA was extracted (RNA Clean & Concentrator5, Zymoresearch), reverse transcribed (Superscript II, Invitrogen), and quantitative Real Time PCR (qRT-PCR) with gene specific primers, using the LightCycler480®system (Roche Applied Science), was performed (Table 1). Data was normalized against *Gapdh* reference gene, with fold induction of transcripts calculated against the indicated control.

**Table 1:** Gene specific primers for qRT-PCR.

Gene	Forward primer (5’ → 3′)	Reverse primer (3’ → 5′)
*mGapdh*	CTC ATG ACC ACA GTC CAT GC	CAC ATT GGG GGT AGG AAC AC
*mTbp*	CTT GAA ATC ATC CCT GCG AG	CGC TTT CAT TAA ATT CTT GAT GGT C
*mLox*	CAC TGC ACA CAC ACA GGG AT	TGT CCA AAC ACC AGG TAC GG
*mLoxl1*	TAC CGA CCC AAC CAG AAT GG	GCT GTG GTA ATG TTG GTG ACA
*mLoxl2*	GAC CTA CAA CCC CAA AGC CT	ACC AAG GGT TGC TCT GGC
*mLoxl3*	TTC ACA GAA GCC ACA GGG TG	CAA CTG ATG CTC CAC CTC AAT
*mLoxl4*	TGG CGT TGC CTG TAT GAA CA	GAT GCT GTG GTA GTG CCT GT
*mSox9*	AAG ACT CTG GGC AAG CTC TGG A	TTG TCC GTT CTT CAC CGA CTT CCT
*mColl2*	ACA CTT TCC AAC CGC AGT CA	GGG AGG ACG GTT GGG TAT CA
*mRunx2*	GGG AAC CAA GAA GGC ACA GA	TGG AGT GGA TGG ATG GGG AT
*mAlpl*	TTG TGC CAG AGA AAG AGA GAG	GTT TCA GGG CAT TTT TCA AGG T
*mBmp2*	AGA TCT GTA CCG CAG GCA CT	GTT CCT CCA CGG CTT CTT C
*mBmp4*	CTC TTC AAC CTC AGC AGC ATC C	TGG CAG TAG AAG GCC TGG TAG C
*mCse*	GCC AGT CCT CGG GTT TTG AA	TTG TGG TGT AAT CGC TGC CT
*mCbs*	AGC AAC CCT TTG GCA CAC TA	CTT ATC CAC CAC CGC CCT G
*m3Mst*	CTG GGA AAC GGG GAG CG	GCT CGG AAA AGT TGC GGG
*mScx*	ACA CCC AGC CCA AAC AG	TCC TTC TAA CTT CGA ATC GCC
*mTnmd*	TCC TGG CCT TAA CTC TAA TTG TC	CTG TTC TGG TTA TGG GAT CAA TTT C
*mCol1a1*	AGG TAT GCT TGA TCT GTA TCT GC	TCC CTC GAC TCC TAC ATC TTC
*mCol31a*	TCC CCT GGA ATC TGT GAA TC	TGA GTC GAA TTG GGG AGA AT

### 3.3 LOX(L) activity

WT and CSE KO tenocytes were plated at 2∗10^5^ cells/cm^2^ and cultured for 24 h in CM. CM consisted of CPP2 (final concentration equivalent to 100 mg/mL calcium [26]) in αMEM Eagle no phenol red. Where indicated, cells were treated with 25 mM STS (Sigma–Aldrich). Total LOX(L) activity, including both extracellular and intracellular fractions and encompassing all LOX isoforms (as no isoform-specific activity assays are currently available) was measured in tenocytes by Lysyl Oxidase Activity Assay Kit (PromoKine). Specific LOX activity was assessed in recombinant human LOX (rhLOX, R&D Systems) incubated with increasing doses of STS (0, 4, 8, 20, 2500 μM) or NaHS (0, 1, 5, 20 μM), using the same kit.

### 3.4 Cytotoxicity (LDH)

Lactate dehydrogenase (LDH) in supernatant was measured using CytoTox-ONE™ Homogeneous Membrane Integrity Assay (Promega) according to the manufacturer's instructions. LDH release (%) was calculated by using the following formula. LDH release (%) = [(value in sample) - (background)]/[(value in Triton X-100-treated sample) - (background)] x100.

### 3.5 Persulfidation

Protein persulfidation was studied using the dimedone-switch method recently described [28]. Murine tenocytes were plated on glass coverslips, 1 mM 4-Chloro-7-nitrobenzofurazan (NBF-Cl, Sigma) was added to cells for 20 min. Cells were fixed in ice-cold methanol, rehydrated in PBS, and incubated with 1 mM NBF-Cl for 1 h at 37 °C. Cells were incubated with Daz2-Cy5.5 (1 mM Daz-2, 1 mM alkyne Cy5.5, 2 mM copper(II)-TBTA, 4 mM ascorbic acid, 20 mM EDTA) for 1 h at 37 °C. Coverslips were mounted in vectashield mounting medium with DAPI and visualized with a Zeiss LSM 800 confocal microscope. Persulfidated (red fluorescence) and total proteins (green fluorescence) were quantified with ImageJ (version 1.8), and ratio of persulfidated/total proteins was calculated.

Alternatively, recombinant human LOX (mybiosource MBS2097305) was incubated with 50 μM H_2_O_2_ and 50 μM NaHS for 15min at 37 °C. 1 mM 4-Chlor-7-nitrobenzofurazan (NBF-Cl, Sigma) was added for 1h 37 °C. The cocktail was incubated with Daz2-Cy5.5 (1 mM Daz-2, 1 mM alkyne Cy5.5, 2 mM copper(II)-TBTA, 4 mM ascorbic acid, 20 mM EDTA) for 30min at 37 °C. Samples were put in Laemmli buffer including 10% b-mercaptoethanol. SDS Page 4-20% was run and visualized with iBright FL1500 Imaging System (Thermo Fischer).

### 3.6 Murine models of tendon calcification

Two murine models of Achilles tendon calcification were used, namely the aging model and the surgically-induced model. This choice was driven by the fact that both aging and surgery/injury are key risk factors for the development of this disease, as previously reported [29]. For aging Achilles tendon calcification, WT and CSE KO mice were sacrificed at 35 weeks of age. This time point was selected because 100% of WT mice exhibit spontaneous Achilles tendon calcification by 35 weeks, thereby permitting the evaluation of genotype-dependent phenotypic differences. For surgically-induced Achilles tendon calcification, tenotomy was performed as recently described and animals were sacrificed at 1, 4, 6 and 16 weeks after surgery [30]. Lower limbs were either kept at −80 °C for biomechanical studies, or fixed in 4% PFA overnight, decalcified for 6 weeks and analyzed by histology and immunohistochemistry.

### 3.7 Mouse tendon microCT-scan

Achilles tendon microCT-scan were acquired using a SkyScan 1076 X-ray μCT scanning system (SkyScan, Belgium): 9 μm resolution, 45 kV, 556 μA, 0.5° rotation step over 360°, 0.2 mm aluminum filter, 780 ms exposure time. 3D reconstruction was performed using NRecon V.1.6.6.0 (Skyscan, Belgium): grey values = 0.0000–0.105867, ring artefact reduction = 10, beam hardening correction = 20%. Quantitative analyses of pathologic calcification (PC), in particular PC Volume (mm^3^), were conducted using CTAnalyzer V.1.10 (SkyScan, Belgium).

### 3.8 Mouse tendon biomechanics

Tendons were mounted in a custom-built system, respecting a physiological angle of 45°, and analyzed as described in Ref. [31]. Briefly, the protocol consisted of a preconditioning phase with a cyclic loading between 0.5% and 1.5% strain at 0.25Hz. Then tendons were stretched to 4% strain for 10 min, to simulate physiological strain. A cyclic loading in a frequency sweep (0.01 Hz, 0.1 Hz, 1 Hz, 5 Hz and 10 Hz) with an amplitude of 0.125% was applied. The same tests were repeated at strain levels of 6% and 8%. Finally, tendons were loaded until failure, and the mode of failure was registered. Data analysis of dynamic and static Young's modulus was done with a customized MATLAB script (R2017a). Dynamic Young's modulus was calculated from the amplitude ratio of the stress-time-curve/strain-time-curve. This represents the tissue's resistance against deflection. Static Young's modulus was calculated from the linear-elastic region of the load-to-failure-curve.

### 3.9 Human tendon histology and quantification of calcification

Human supraspinatus tendons were obtained from 16 patients undergoing arthroscopic surgery for treatment of calcific tendinopathy (52.84 ± 10.75 years). After resection, tendons were fixed in 4% PFA for calcification and immunohistochemical analysis. Quantitative measurement of tendon calcification was performed using ImageJ, by applying the same thresholds to all samples. The percentage of calcification over the whole tendon area was calculated. Tendon samples were grouped into low (0-3% of total tissue, age: 47.79 ± 13.19 years), medium (3-20% of total tissue, age: 57.16 ± 12.88) and high (>20% of total tissue, age: 54 ± 7.65 years) tendon calcification (Fig. S2A).

### 3.10 Mouse and human tendon histological and immunohistochemical analysis

Stainings were performed on paraffin sections. Murine CSE expression was evaluated using an anti-CSE rabbit polyclonal antibody (Proteintech, CSE 12217-1-AP), human CSE expression using an anti-CSE rabbit polyclonal antibody (Sigma–Aldrich, HPA-023300). An arbitrary score from 0 (0% positive area) to 4 (>75 positive area) was given to three fields for each sample (Fig. S2B for human and Fig. S3A for mice) and the mean values plotted in the graphs. Murine and human LOX expression was evaluated using an anti-LOX rabbit polyclonal antibody (Proteintech, 17958-1-AP). For human tendons, an arbitrary score from 0 (0% positive area) to 4 (>75 positive area) was given to three fields for each sample (Fig. S2C) and the mean values plotted in the graphs. For murine Achilles tendons, an arbitrary score from 0 (no positivity) to 3 (high positivity) was given, excluding the adjacent tissues for unbiased scoring (Fig. S3B). Murine COL II and COL X expression were evaluated using an anti-COL II mouse monoclonal antibody (Abcam, ab79127) and an anti-COL X rabbit polyclonal antibody (Genetex GTX37732), respectively. Quantitative measurement of COL II and COL X expression was performed using ImageJ, by applying the same upper and lower bounds of the threshold utility to all samples. These areas were used for calculating the percentage of COL II and COL X expression over the whole tendon area. Picrosirius red staining was performed on murine tendon sections.

### 3.11 Bioinformatics analysis

Gene expression profile GSE181172 obtained from a published study [32] was downloaded from the Gene Expression Omnibus (www.ncbi.nlm.nih.gov/geo/). The study included data from four mice with surgically induced Achilles tendon injury and four sex- and age-matched sham-operated mice, whose tendons were homogenized for RNA extraction and sequenced by Illumina HiSeq 2500. Volcano plots and heatmap were obtained using Seurat (version 4.3.0.1) [33] in R software (version 4.1), while fold change results were obtained from Ref. [32], and was confirmed by our analysis with Seurat (function FindMarkers with DESeq2 test).

### 3.12 Study approval

Tendon experiments in mice were carried out with permission of animal rights protection authorities in accordance with the German National Institutes of Health guidelines (authorization LANUV NRW, AZ 84-02.04.2015.A310) or approved by the “Service de la Consommation et des Affaires Vétérinaires du canton de Vaud,” Switzerland (authorization VD3737). Mice were housed at a constant temperature (21 °C) and humidity (65%), and under a 12h light/dark cycle with free access to water and food. All efforts were made to minimize suffering. Human tendon samples were obtained by the institutional Review Board of the Faculty of Medicine of the Otto-von-Guericke University (IRB No 84/19) and following patients written informed consent.

### 3.13 Statistical analysis

For *in vitro* experiments, values represent means ± SD of replicates from one representative experiment out of at least two experiments. For *in vivo* experiments, 5 to 7 mice per group were used. WBs represent means ± SD of replicate experiments (n = 3 to 5). Data was analyzed with GraphPad Prism (Version 9), San Diego, CA. Variation between datasets was assessed using appropriate statistical tests. For comparisons between two groups, the Student's t-test or Mann–Whitney U test was applied for parametric or nonparametric data, respectively. For multiple comparisons, one-way or two-way ANOVA was performed, with p-values adjusted using the Tukey correction when comparing all experimental groups with each other, and the Dunnett correction when comparing all experimental groups to a single control. For biomechanical data, the Greenhouse–Geisser correction was applied because the assumption of sphericity was violated (i.e., the variances of the differences between repeated measures were unequal). Differences were considered statistically significant at ∗p < 0.05, ∗∗p < 0.01, ∗∗∗p < 0.001, ∗∗∗∗p < 0.0001.

## 4 Results

### 4.1 CSE expression negatively correlates with LOX and calcification in human calcific tendinopathy and in an RNA-seq analysis of injured murine tendons

**Fig. 1:**
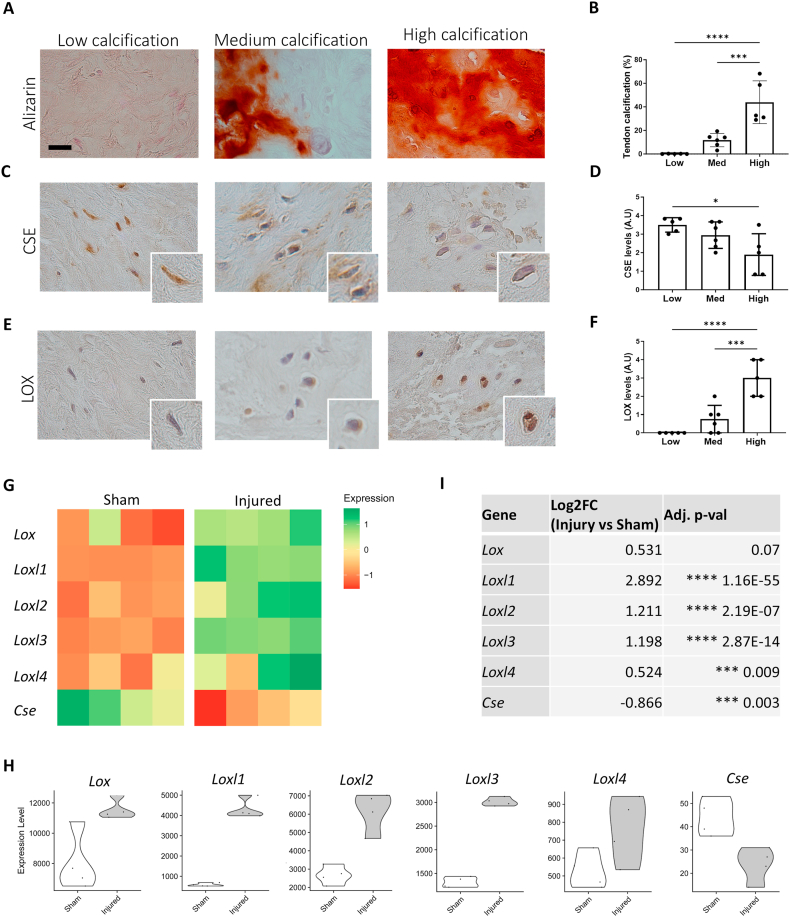
CSE negatively correlates with LOX and calcification in human and murine calcific tendinopathy (A) Alizarin Red staining in tendons from individuals with low and high calcific tendinopathy. One representative picture from one patient per group is shown. Scale bars 20 μm. (B) Graphs show % of tendon calcification over total tissue area. Alizarin red staining was quantified by Image J as % of total tissue area. One-way ANOVA, n = 16. (C) CSE immunohistochemistry in tendons from individuals with low and high calcific tendinopathy. One representative picture from one patient per group is shown. Scale bars 20 μm. (D) Graphs show quantification of CSE (A.U). For each patient three fields were scored per explant and the mean plotted. One-way ANOVA, n = 16. (E) LOX immunohistochemical staining in tendons from individuals with low and highly calcified tendons. One representative picture from one patient per group is shown. Scale bars 20 μm. (F) Graphs show quantification of LOX levels (A.U). For each patient three fields were scored per explant and the mean plotted. One-way ANOVA, n = 16. (G) Heatmap and (H) violin plots of the reported genes resulting from RNA-seq analysis of sham and injured murine tendons. (I) Results from differential gene expression analysis of injury vs sham operated murine tendons. n = 4. ∗∗p < 0.01, ∗∗∗p < 0.001, ∗∗∗∗p < 0.0001.

Human tendons from 16 patients with calcific tendinopathy were classified as having low, medium, or high calcification, based on the extent of alizarin red staining (Fig. 1A and B). CSE was prominently expressed in tendons with low calcification but significantly decreased in highly calcified samples (Fig. 1C). Quantitatively, CSE expression was high in the low calcified tendon samples, a bit lower in the moderately calcified tendon and significantly diminished in highly calcified tendons (Fig. 1D). This was confirmed by a significant negative correlation between CSE levels and the degree of tendon calcification throughout all human samples (Fig. S4A). On consecutive sections, we observed minimal LOX levels in tendons with low calcification, where tenocytes exhibited a typical fibroblast-like phenotype (Fig. 1E). Conversely, high LOX levels were found in highly calcified tendons, particularly in chondrocyte-like cells (Fig. 1E). These observations were confirmed by quantifications (Fig. 1F). Overall, a significant positive correlation between LOX levels and the degree of tendon calcification was found throughout all human samples and Fig. S4B).

Analysis of previously published RNA sequencing data from four sham-operated and four partially transected murine Achilles tendons [32] revealed an increase of *Lox(l)* genes in injured tendons, together with decreased *Cse* expression (Figs. 1G and 8H). Highly significant modulation of these genes was confirmed by differential gene expression analysis performed by both Cho et al. [32] and us (Fig. 1I).

### 4.2 Hydrogen sulfide (H_2_S) inhibits murine tenocyte calcification

**Fig. 2:**
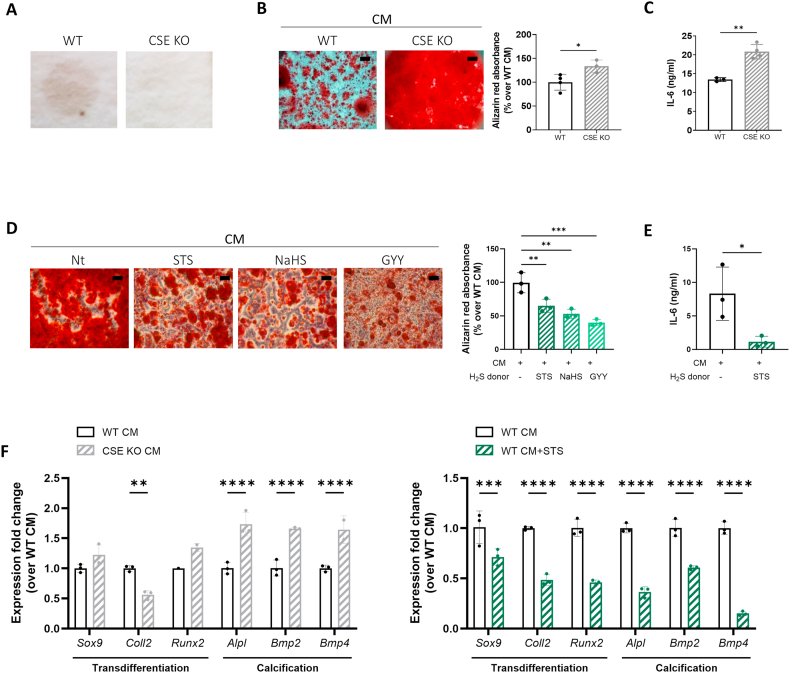
H_2_S inhibits murine tenocyte calcification (A) Lead acetate analysis of endogenous H_2_S production by WT and CSE KO tenocytes. (B) Alizarin red staining of WT and CSE KO tenocytes cultured in calcification medium (CM) for 24 h. Graph represents Alizarin red quantification in the cell monolayer. Unpaired t-test, n = 3-4. (C) IL-6 secretion in cell supernatant of WT and CSE KO tenocytes cultured as in (B). Unpaired t-test, n = 3 4. (D) Alizarin red staining of WT tenocytes cultured for 24 h in calcification medium and treated or not with H_2_S-donors (STS, NaHS, GYY). Graph represents Alizarin red quantification in the cell monolayer. Scale bars 500 μm. One-way ANOVA, n = 3. (E) IL-6 secretion in cell supernatant of tenocytes cultured as in (D). Unpaired t-test, n = 3. (F) qRT-PCR analysis of the indicated genes in WT and CSE KO murine tenocytes cultured for 24h in CM and treated or not with STS. Expression was normalized over WT CM (=1). Two-way ANOVA, n = 3. ∗p < 0.05, ∗∗p < 0.01, ∗∗∗p < 0.001, ∗∗∗∗p < 0.0001.

After confirming effective gene knock-down in CSE knockout (CSE KO) tenocytes (Fig. S1B), we used the lead-acetate method to show that CSE KO tenocytes functionally produced less H_2_S compared to wild-type (WT) control tenocytes, as indicated by a fainter brown signal (Fig. 2A). When cultured in calcification medium (CM), CSE KO tenocytes exhibited a significantly increased level of calcification compared to WT cells, demonstrated by Alizarin red staining of cell monolayers and corresponding quantification (Fig. 2B). We additionally found that secretion of IL-6, a known pro-calcification cytokine [34], was significantly higher in CSE KO tenocytes (Fig. 2C). The levels of two additional pro-inflammatory cytokines (IL-1β and TNF-α) was assessed in cell supernatant, but they resulted undetectable. Consistently, H_2_S donors (STS, NaHS, or GYY) significantly suppressed crystal formation (Fig. 2D) as well as IL-6 release (Fig. 2E) in WT tenocytes. These effects were not due to cytotoxicity of the H_2_S donors (Fig. S5A), as the percentage of cell toxicity measured by LDH release in cell supernatant was comparable between conditions.

We explored whether the transdifferentiation of tenocytes into calcifying chondrocyte-like cells could be a contributing factor to increased calcification in CSE KO tenocytes. Although no changes in chondrogenic genes (*Sox9, Coll2, Runx2*) were found when comparing WT and CSE KO tenocytes, CSE KO cells displayed upregulation of calcification-related genes (*Alpl, Bmp2, Bmp4*) (Fig. 2F, left). By contrast, STS significantly diminished the expression of both chondrogenic and calcification genes (Fig. 2F, right).

### 4.3 LOX(L) inhibition diminishes murine tenocyte calcification

**Fig. 3:**
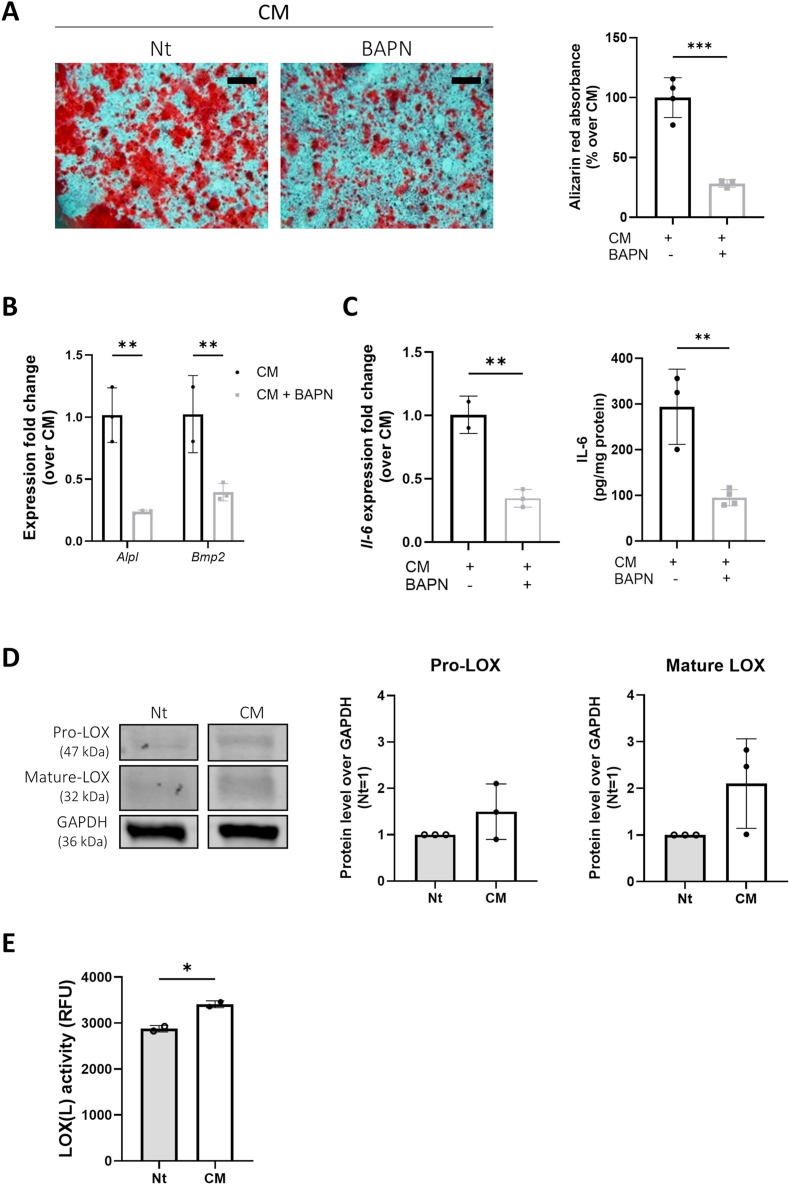
LOX(L) inhibition diminishes murine tenocyte calcification (A) Alizarin red staining of WT tenocytes cultured for 14 days in CM and treated or not with BAPN. Graph represents Alizarin red quantification in the cell monolayer. Unpaired t-test, n = 3-4. Scale bar 500 μm. (B) qRT-PCR analysis of the indicated genes in tenocytes as in (A). Expression was normalized over CM (=1). Two-way ANOVA, n = 3. (C) qRT-PCR analysis of IL-6 gene and IL-6 secretion by tenocytes cultured as in (A). Unpaired t-test, n = 3-4. (D) WB for pro-LOX, mature-LOX, and β-ACTIN in tenocytes cultured 14 days in Nt medium or CM. Graphs represent the ratio of band intensity of CM over Nt, normalized over β-ACTIN. Unpaired t-test, n = 3. (E) LOX(L) activity measured in WT tenocytes cultured or not in CM. Unpaired t-test, n = 2. ∗p < 0.05, ∗∗p < 0.01, ∗∗∗p < 0.001.

Since LOX(L) inhibition has been shown to protect against calcification in chondrocytes [19,35] and vascular smooth muscle cells [20], we investigated whether similar effect could be observed in tenocytes. Our findings indicate that the LOX(L) inhibitor BAPN significantly reduced calcium-containing crystals (Fig. 3A) and the expression of calcification-related genes *Alpl* and *Bmp2* in tenocytes (Fig. 3B). This reduction was not due to cytotoxic effects of BAPN (Fig. S5B), as BAPN treated cells displayed similar percentage of cell toxicity when compared to untreated cells. Additionally, BAPN inhibited IL-6 expression at both gene and protein levels (Fig. 3C). Furthermore, we proved that calcification stimuli tended to augment LOX (pro- and mature forms) levels (Fig. 3D), and significantly induced LOX activity (Fig. 3E).

### 4.4 Endogenous and exogenous H_2_S suppresses LOX(L) activity

**Fig. 4:**
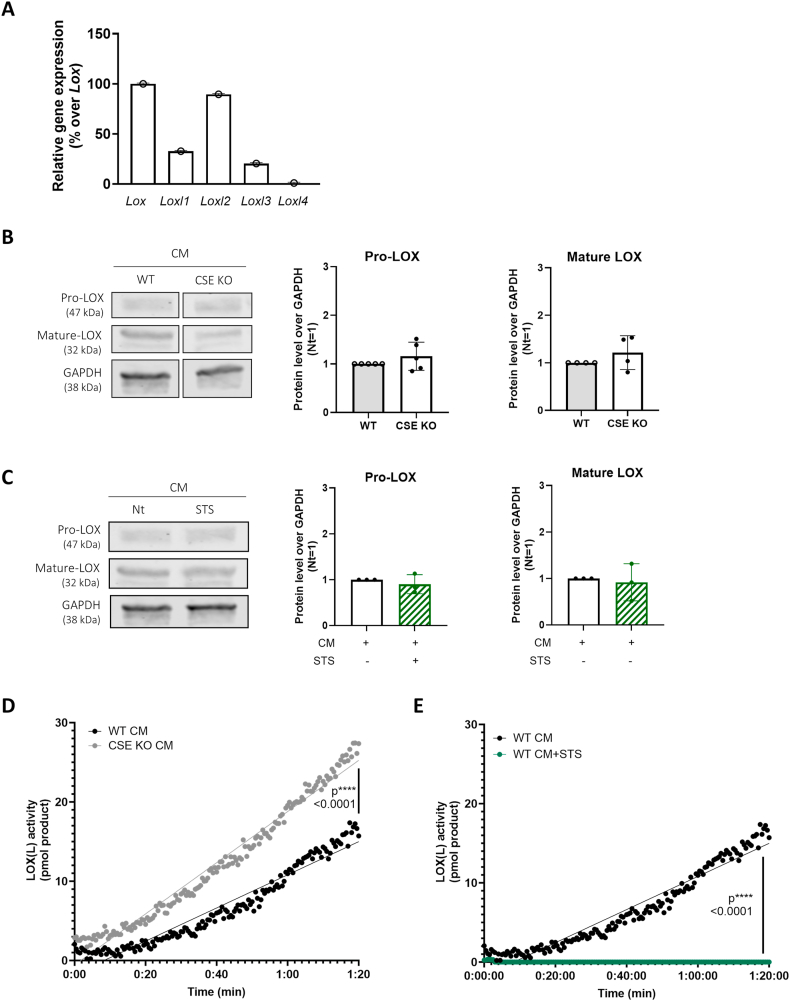
H_2_S inhibits LOX(L) activity (A) qRT-PCR analysis of the indicated genes in murine tenocytes. Expression was normalized over Lox expression (=100%). Two-way ANOVA, n = 3. (B) WB for pro-LOX, mature-LOX, and GAPDH in WT and CSE KO tenocytes cultured 24 h in CM. Graphs represent the ratio of band intensity of CSE KO over WT, normalized over housekeeping. Unpaired t-test, n = 3. (C) WB for pro-LOX, mature-LOX, and GAPDH in WT tenocytes cultured 24 h in CM in presence or absence of STS. Graphs represent the ratio of band intensity of STS/NaHS over Nt, normalized over housekeeping. Unpaired t-test, n = 3. (D) Kinetic of LOX(L) activity measured in WT and CSE KO tenocytes cultured for 24 h in CM. Simple linear regression. (E) Kinetic of LOX(L) activity measured in WT tenocytes cultured for 24 h in CM and treated or not with STS. Simple linear regression.

Given the similar anti-calcification effect elicited by H_2_S and by the LOX(L) inhibitor BAPN on tenocytes, we hypothesized that H_2_S might function as a LOX(L) inhibitor. Amongst the five *Lox(l)* genes, we identified *Lox* as the most highly expressed in tenocytes (Fig. 4A). We therefore examined its modulation by H_2_S. When comparing WT and CSE KO tenocytes cultured in CM, we found no difference in pro-LOX and mature LOX levels (Fig. 4B). However, LOX(L) activity was markedly increased in CSE-deficient tenocytes (Fig. 4D). In line, although exogenous H_2_S (STS) did not affect LOX protein levels (Fig. 4C), it significantly inhibited LOX(L) activity (Fig. 4E).

### 4.5 H_2_S inhibits LOX via persulfidation

**Fig. 5:**
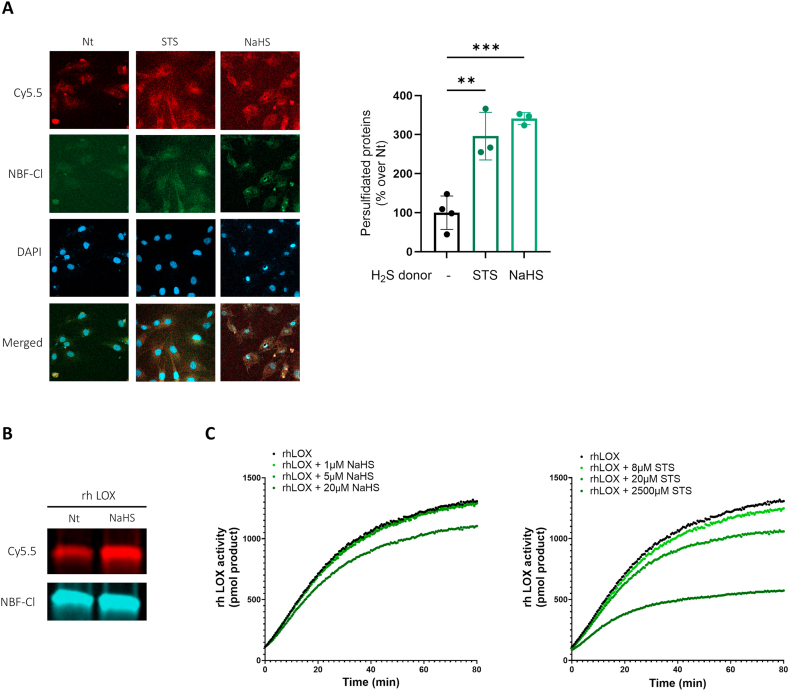
H_2_S inhibits LOX(L) activity via persulfidation (A) Confocal microscopy images and quantification of intracellular persulfidated proteins assessed by the dimedone-switch method: Cy5.5-labled Daz2 (red), normalized to NBF-adducts fluorescence (green), in WT tenocytes treated or not with STS or NaHS for 24h. One-way ANOVA, n = 3-4. Scale bar 50 μm. (B) rhLOX persulfidation assessed by the dimedone-switch method, on gel. Top row: Cy5.5-labled Daz2 signal; middle row: NBF adducts. (C) Human recombinant LOX activity upon treatment with increasing doses of H_2_S-donors (NaHS and STS). ∗∗p < 0.01, ∗∗∗p < 0.001∗∗∗∗, p < 0.0001.

We next investigated the potential mechanisms underlying H_2_S-mediated inhibition of LOX. The dimedone-switch method revealed that murine tenocytes treated with STS or NaHS exhibited a threefold increase in persulfidated proteins (Fig. 5A, red staining and graph). Additionally, we demonstrated that NaHS induced persulfidation of recombinant human LOX (Fig. 5B, red bands). Notably, persulfidation was accompanied by a dose-dependent inhibition of rhLOX enzymatic activity following NaHS or STS treatment (Fig. 5C).

### 4.6 CSE expression negatively correlates with LOX and calcification in murine surgery-induced tendinopathy

**Fig. 6:**
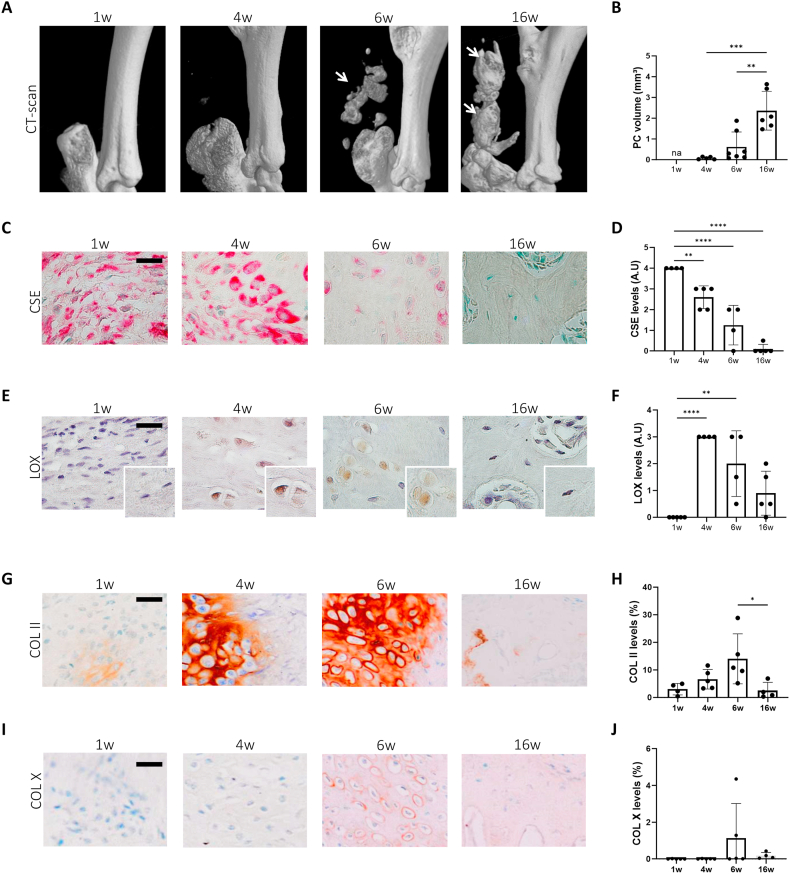
CSE inversely correlates with LOX and calcification in surgery-induced murine calcific tendinopathy (A) Representative micro-CT scan images of WT murine Achilles tendons at 1, 4, 6 and 16 weeks after tenotomy. White arrows show increased calcification over time. (B) Graph shows CTAnalyzer quantitative analysis of pathologic calcification (PC) volume (mm3) over time. One-way ANOVA, n = 5-7. (C) Representative CSE immunohistochemical staining of tendons from (A) and (D) CSE quantification in arbitrary units. One-way ANOVA, n = 4-5. Scale bars 20 μm. (E) Representative LOX immunohistochemical staining of tendons from (A). Scale bars 20 μm. (F) LOX quantification in arbitrary units. One-way ANOVA, n = 4-5. (G) Representative COL II immunohistochemical staining of tendons from (A). Scale bars 20 μm. (H) COL II quantification in % over tissue area. One-way ANOVA, n = 4-5. (I) Representative COL X immunohistochemical staining of tendons from (A). Scale bars 20 μm. (J) COL X quantification in % over tissue area. One-way ANOVA, n = 4-5. ∗p < 0.05, ∗∗p < 0.01, ∗∗∗p < 0.001, ∗∗∗∗p < 0.0001.

In murine surgery-induced calcific tendinopathy, microCT-scan reconstructions revealed an increase of pathologic calcification (PC) of tendons over time (Fig. 6A white arrows, and Fig. 6B). Consistent with our *in vitro* findings, increased calcification was associated with a gradual loss of CSE expression (Fig. 6C and D) and a concurrent increase in LOX expression (Fig. 6E). LOX levels peaked at 4 and 6 weeks (Fig. 6F), primarily in roundish cells resembling chondrocytes, as depicted in the magnified squares (Fig. 6E). Collagen type II immunohistochemistry confirmed that the predominant fibroblast-like tenocytes present at 1 week transdifferentiated into chondrocytes by 4-6 weeks (Fig. 6G and E). Collagen type II almost disappeared at 16 weeks, when tissue became ossified (Fig. 6G and E). At later stages (6-16 weeks), cells began expressing collagen type X, a typical marker of calcification and bone formation (Fig. 6I and J).

### 4.7 Aged CSE-deficient mice show increased tendon calcification, LOX expression, and altered biomechanics

**Fig. 7:**
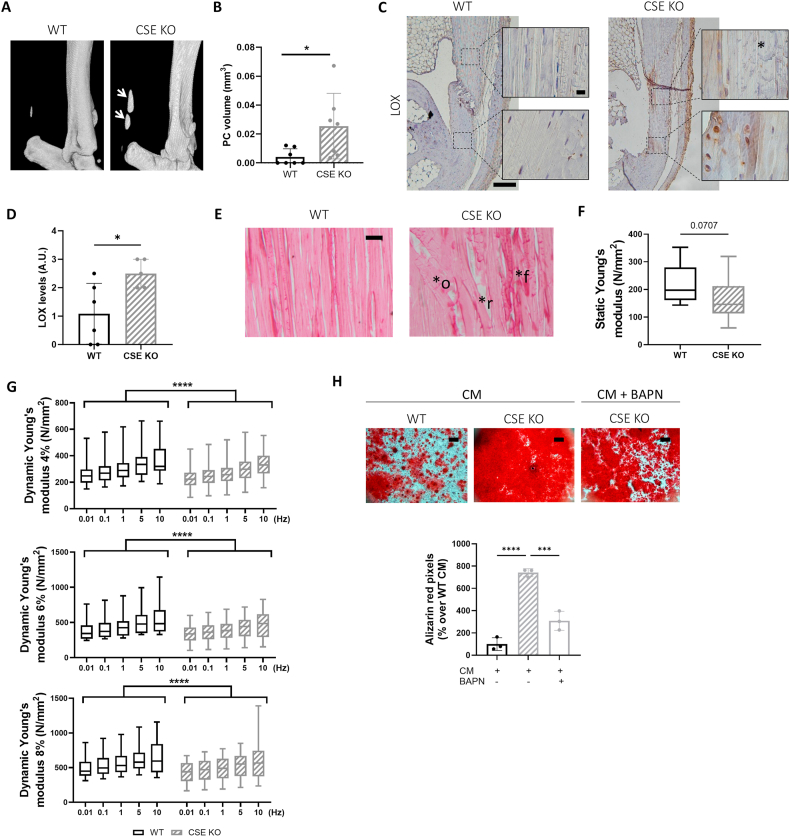
CSE deficiency associates with increase of LOX, calcification and impaired tendon mechanical properties in aged mice with calcific tendinopathy (A) Representative micro-CT scan images of WT and CSE KO murine Achilles tendons at 35 weeks of age. White arrows show increased calcification in CSE KO mice. (B) Graph shows CTAnalyzer quantitative analysis of PC volume (mm3) in WT and CSE KO mice. Mann–Whitney test, n = 7. (C) Representative LOX immunohistochemical staining of tendons from (A). Left picture scale bare 200 μm, right picture scale bar 10 μm. (D) Graphs show corresponding LOX scoring (A.U.). Unpaired t test, n = 5-6. ∗p < 0.05. (E) Representative Picrosirius red staining for collagen fibers of tendons from (Fig. 6A) ∗o = organization; ∗r = rupture; ∗f = fibrosis. Scale bar 10 μm. (F) Static and (G) dynamic Young's modulus (N/mm^2^) obtained from pulling tests of WT and CSE KO Achilles tendons at 35 weeks of age (4,6 and 8% elongation). Unpaired t test and Two-way ANOVA, n = 10-18. ∗∗∗∗p < 0.0001. (H) Alizarin red staining of WT and CSE KO tenocytes cultured for 14 days in CM and treated or not with BAPN. Graph represents Alizarin red quantification in the cell monolayer. One-way ANOVA, n = 3. Scale bars 500 μm ∗∗∗p < 0.001, ∗∗∗∗p < 0.0001.

In aged mice, we observed markedly increased spontaneous calcification of the Achilles tendon in CSE KO mice compared to WT littermates (Fig. 7A white arrows, and Fig. 7B).

Concomitantly, LOX level was more elevated in CSE deficient mice (Fig. 7C and D) and was predominantly present in roundish chondrocyte-like cells and their surrounding extracellular matrix (Fig. 7C, magnified rectangles). Additionally, tendon fibrils in CSE KO appeared disorganized compared to the elongated, regular fibrils in WT mice (Fig. 7C, black asterisks). Picrosirius red staining confirmed that collagen fibrils in WT mice were structurally homogenous, well aligned and properly oriented while fibrils of CSE deficient mice appeared with altered orientation (Fig. 7E, ∗o), signs of rupture (Fig. 7E, ∗r), and fibrosis (Fig. 7E, ∗f).

For functional analysis of tendons, we performed biomechanical pulling tests on WT and CSE KO tendons. Static Young's modulus, representing the resistance to tendon rupture, was reduced in CSE KO (Fig. 7F). Furthermore, at physiologic (4%), over-physiologic (6%) and pathologic (8%) deflection, CSE KO tendons exhibited a highly significant reduction of dynamic Young's modulus, indicating decreased resistance to strain (Fig. 7G). The significantly reduced dynamic Young's modulus observed in CSE KO tendons indicates that these tendons are mechanically weaker and less resistant to deformation under cyclic loading. This reduction likely reflects a combination of structural alterations, including pathologic calcification, disorganized collagen fibrils, and altered fibril alignment (Fig. 7A, B, E). While reduced collagen cross-linking could theoretically contribute to decreased stiffness, this is unlikely in CSE KO tendons given the higher LOX levels (Fig. 7C) and increased LOX(L) activity previously (Fig. 4D). Finally, to support the conclusion that the increased tendon calcification in CSE knockout mice is LOX-dependent, we performed a rescue experiment. Due to restrictions on animal experimentation, this could not be conducted *in vivo*; instead, we carried out the experiment in calcifying tenocytes *in vitro*. The results show that inhibition of LOX in CSE knockout tenocytes almost completely abolishes the increased calcification observed compared to wild-type cells.

## 5 Discussion

In this study, we examined the roles of lysyl oxidase (LOX) and hydrogen sulfide (H_2_S) in calcific tendinopathy (CT) using a reverse translational approach, addressing the ethical and practical constraints of mechanistic studies in humans. Our investigation was driven by the observation that, in human tendon samples, reduced expression of cystathionine γ-lyase (CSE), one of the enzymes responsible for endogenous H_2_S production, was inversely correlated with the extent of tendon calcification. In contrast, LOX expression showed a positive correlation with calcification severity. Based on these findings, we hypothesized that reduced CSE expression in human tendons promotes LOX-driven calcification.

To test this hypothesis, we first used tenocytes isolated from CSE KO mice. As expected, these cells produced markedly less H_2_S and showed increased calcification compared WT tenocytes. Notably, supplementation with exogenous H_2_S, via donors with different release kinetics such as sodium thiosulfate (STS), sodium hydrosulfide (NaHS), or GYY4137, reversed the calcification phenotype *in vitro*.

Next, we confirmed the inverse relationship between CSE levels and LOX/calcification in two murine models that closely mirror the clinical progression of human CT: a surgery-induced tenotomy model and an aging model. In the tenotomy model, CSE levels progressively declined in parallel with increased LOX/calcification. Similarly, aged CSE-KO mice showed greater LOX/calcification in tendons than age-matched WT controls.

Further mechanistic analyses revealed that exogenous H_2_S inhibits the transdifferentiation of tenocytes into chondrocyte-like cells both *in vitro* and *in vivo*. Expression of transdifferentiation markers (Sox9, Col2a1, Runx2) and calcification markers (Alpl, Bmp2, Bmp4) was significantly downregulated following H_2_S treatment. These findings are consistent with previous studies showing that exogenous H_2_S reduces vascular calcification [9,36] and mitigates cartilage damage and crystal deposition in osteoarthritis models [5]. In line, reduced endogenous H_2_S levels, due to deficiency in any of the H_2_S-producing enzymes (3-MST [8], CBS, or CSE [6]), increase calcification in chondrocytes, and an inverse correlation between 3-MST or CSE expression and calcification severity has been reported in human osteoarthritis cartilage [6,8]. Altogether, these data support a unifying paradigm in which endogenous H_2_S is a key regulator of calcification, and maintaining its physiological levels is essential for preventing pathological mineralization and preserving tissue integrity. All these findings appear in contrast with the study by Yuan et al. [10], reporting that exogenous H_2_S accelerates trauma-induced heterotopic ossification in tendons. However, several methodological and conceptual differences distinguish their study from ours. First, they focus primarily on ossification, whereas our study investigates earlier upstream events related to tendon calcification. Second, they employed a high dose of NaHS (5 mg/kg daily for 14 days), which may exert toxic effects, given the well-established biphasic nature of H_2_S signaling (cytoprotective at low concentrations and potentially deleterious at high concentrations). Moreover, endogenous H_2_S production in their study was assessed exclusively using propargylglycine, a relatively non-selective pharmacological inhibitor of CSE, whereas our approach relies on genetic deletion of CSE in cells and mice, providing a more specific model. In addition, their murine model consisted of tendon surgery combined with burn injury, a setting that promotes trauma-induced heterotopic ossification. In contrast, our models include both surgery/trauma-induced and spontaneous (aging-associated) calcification, better reflecting degenerative calcific tendinopathy. Finally, the human samples analyzed by Yuan et al. were derived from ossified tendons following surgery or from knee tendons, which are not directly comparable to our supraspinatus tendon samples obtained during arthroscopic treatment of primary calcific tendinopathy, independent of prior surgical intervention.

In the current study we additionally clarified that LOX enzyme may promote tendon calcification. LOX has been recently implicated in vascular calcification as LOX-overexpressing mice displayed aberrant calcification [20] while LOX inhibition by BAPN reduced vascular calcification [21]. Our previous work in chondrocytes yielded similar findings, with BAPN effectively preventing crystal deposition in cartilage cells [19]. Potential mechanisms by which LOX may drive tendon calcification include induction of calcification triggers such as reactive oxygen species (ROS) [37] and inflammation [34]. LOX(L) generates ROS as a by-product of its enzymatic activity [18], and mitochondrial ROS can stimulate LOX-mediated collagen cross-linking [38]. Furthermore, intracellular LOX translocation has been shown to upregulate the pro-calcifying cytokine IL-6 in scleroderma [17]. Consistent with this, we found that BAPN treatment reduced IL-6 secretion in tenocytes.

The major finding of this study is the identification of a CSE(H_2_S)–LOX–calcification regulatory axis, where endogenous H_2_S serves as a key physiological inhibitor of LOX-driven calcification. In tenocytes isolated from CSE KO mice, LOX activity was significantly elevated compared to WT cells. Consistently, treatment with H_2_S donors suppressed LOX activity in calcifying tenocytes. This inverse relationship between CSE and LOX/calcification was observed across both aging and surgically induced calcific tendinopathy (CT) models, as well as in human CT samples. Transcriptomic analysis from an independent CT model further confirmed this pattern, revealing decreased CSE mRNA alongside elevated *Lox* gene expression.

One potential mechanism by which H_2_S exerts its effects is through persulfidation, a post-translational modification in which cysteine residues are converted to persulfides, potentially altering protein function [3,39,40]. LOX and LOXL2 contain multiple cysteine residues and disulfide bridges in their active sites, which are essential for enzymatic activity and may be susceptible to disruption by H_2_S [41]. We observed increased levels of persulfidated proteins in tenocytes treated with H_2_S donors. Further evidence came from a cell-free assay, in which recombinant human LOX exhibited enhanced persulfidation and reduced enzymatic activity following incubation with an H_2_S donor. Other LOX family members, which share the catalytic domain, may also be regulated by the CSE/H_2_S axis.

In conclusion, our findings identify LOX as a key driver of tendon pathologic calcification and establish a mechanistic link between reduced H_2_S production and increased LOX activity. These results position LOX as a promising therapeutic target for the treatment of calcific tendinopathy other disorders characterized by pathological calcification. Potential therapeutic strategies include the use of LOX inhibitors, such as small molecules or neutralizing antibodies currently under preclinical development, as well as approaches aimed at restoring H_2_S homeostasis. These may involve recently discovered positive allosteric modulators that selectively stimulate endogenous CSE activity [42] or the use of H_2_S donors. However, translating H_2_S-based therapies to the clinic presents several challenges. Systemic or local H_2_S supplementation carries the risk of off-target effects, including hypotension, interference with mitochondrial respiration, and modulation of inflammatory responses, highlighting the importance of careful dose titration. Achieving therapeutically effective yet safe H_2_S levels may require controlled-release formulations, tissue-targeted delivery, or strategies that selectively stimulate endogenous CSE activity rather than relying solely on exogenous donors. Furthermore, inter-individual differences in H_2_S metabolism, comorbid conditions, and concomitant medications may affect both efficacy and safety, underscoring the complexity of clinical translation. Overall, controlled and targeted delivery would minimize the risk of excessive H_2_S supplementation while maximizing tendon-protective effects.

## Data statement

All data are available in the main text or the supplementary materials. Raw data are available upon request to Sonia.Nasi@chuv.ch.

## Author contribution

Conceptualization: SN, NB. Data curation; Formal analysis; Investigation; Methodology: SN, IB, EF, DE, DK, PM. Visualization: SN, IB, EF, DE, DK. Funding acquisition: SN, DK, RS. Project administration: SN. Supervision: SN, NB. Writing – original draft: SN, IB, EF, GC. Writing – review & editing: SN, NB, DK, TP, RS, GC.

## AI statement in scientific writing

No generative artificial intelligence (AI) or AI-assisted technologies were used in the preparation.

## Funding sources

This work was supported by the Suisse National Science Foundation (SNSF) [Grant n°320030_204524.1] to Sonia Nasi and by the Deutsche Forschungsgemeinschaft DFG [Grant number Sta 650/13-1] to Richard Stange and Daniel Kronenberg.

## Declaration of competing interest

The author(s) have no conflicts of interest relevant to this article.
