# Supplementary material for: Hydrogen sulfide generated by cystathionine gamma lyase inhibits lysyl oxidase and protects against calcific tendinopathy
Source: J Orthop Translat. 2026 Mar 25;57:101082. doi: 10.1016/j.jot.2026.101082 (PMC13053855; doi:10.1016/j.jot.2026.101082)
Supplement: Multimedia component 1 [file mmc1.docx]

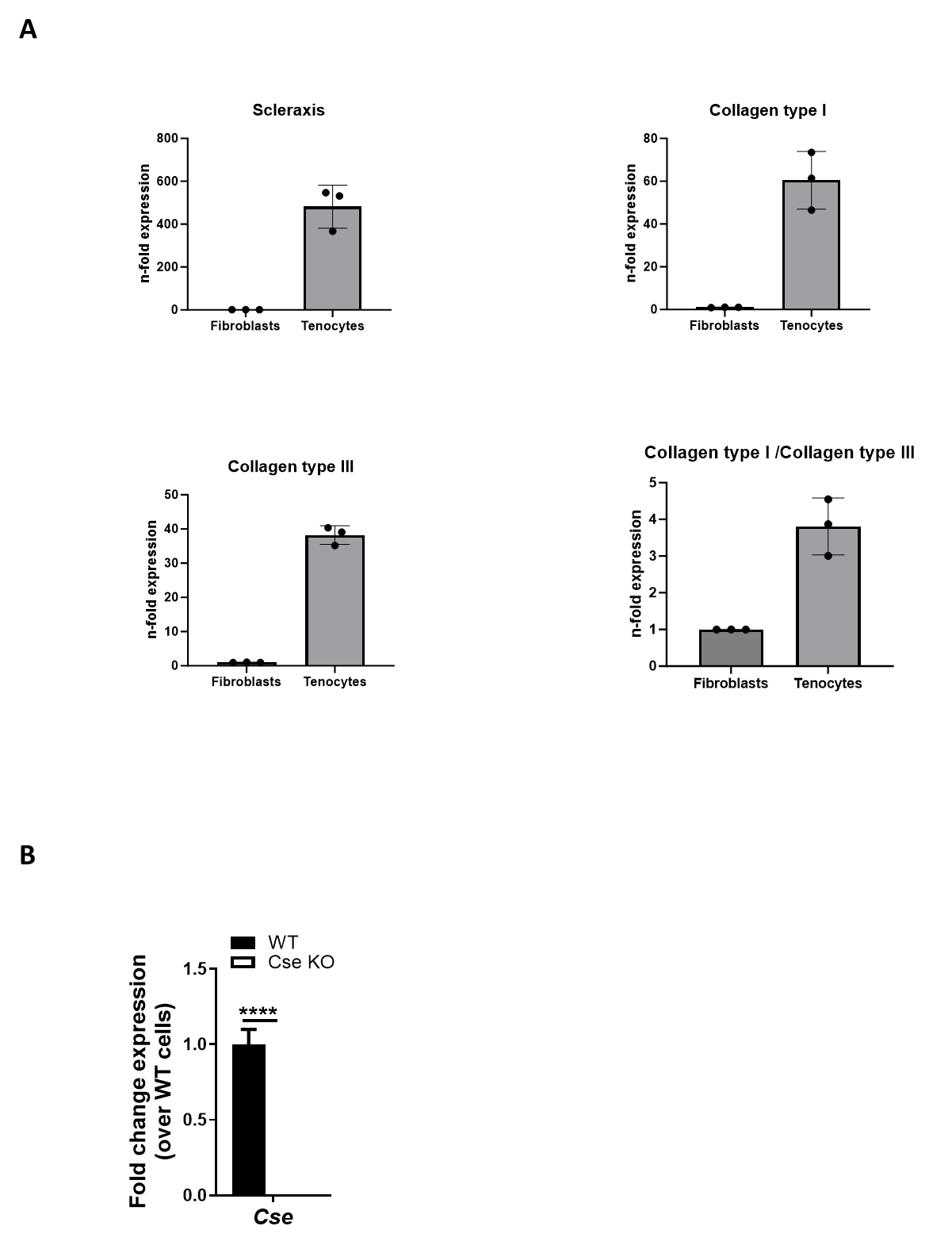


**Fig. S1:** qRT-PCR for the indicated genes in NIH3T3 and in primary murine tenocytes


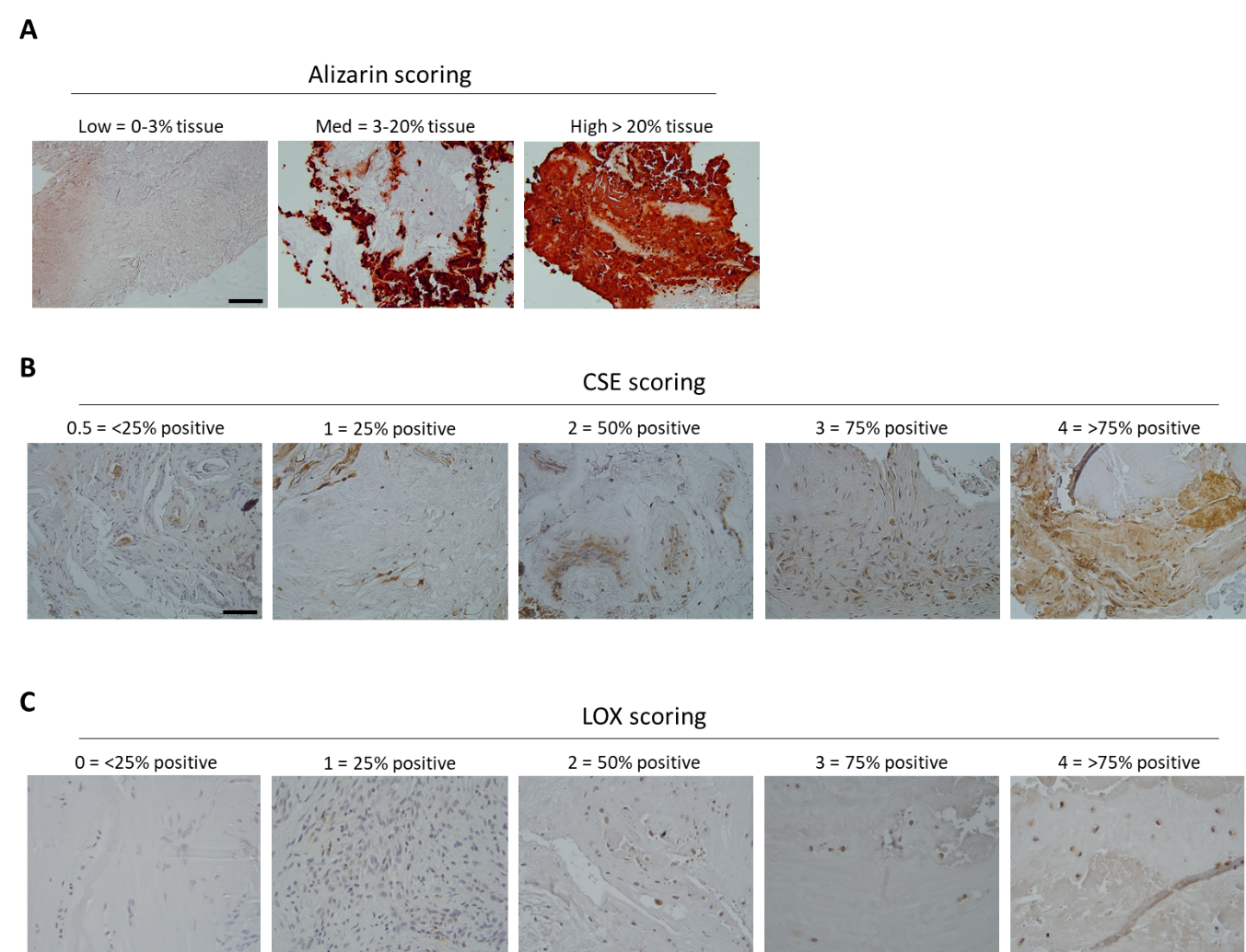


Fig. S2: Scoring system in human tendons for: (A) Alizarin red staining, (B) CSE expression, (C) LOX expression. Scale bars (A) 200µm, (B-C) 50µm.


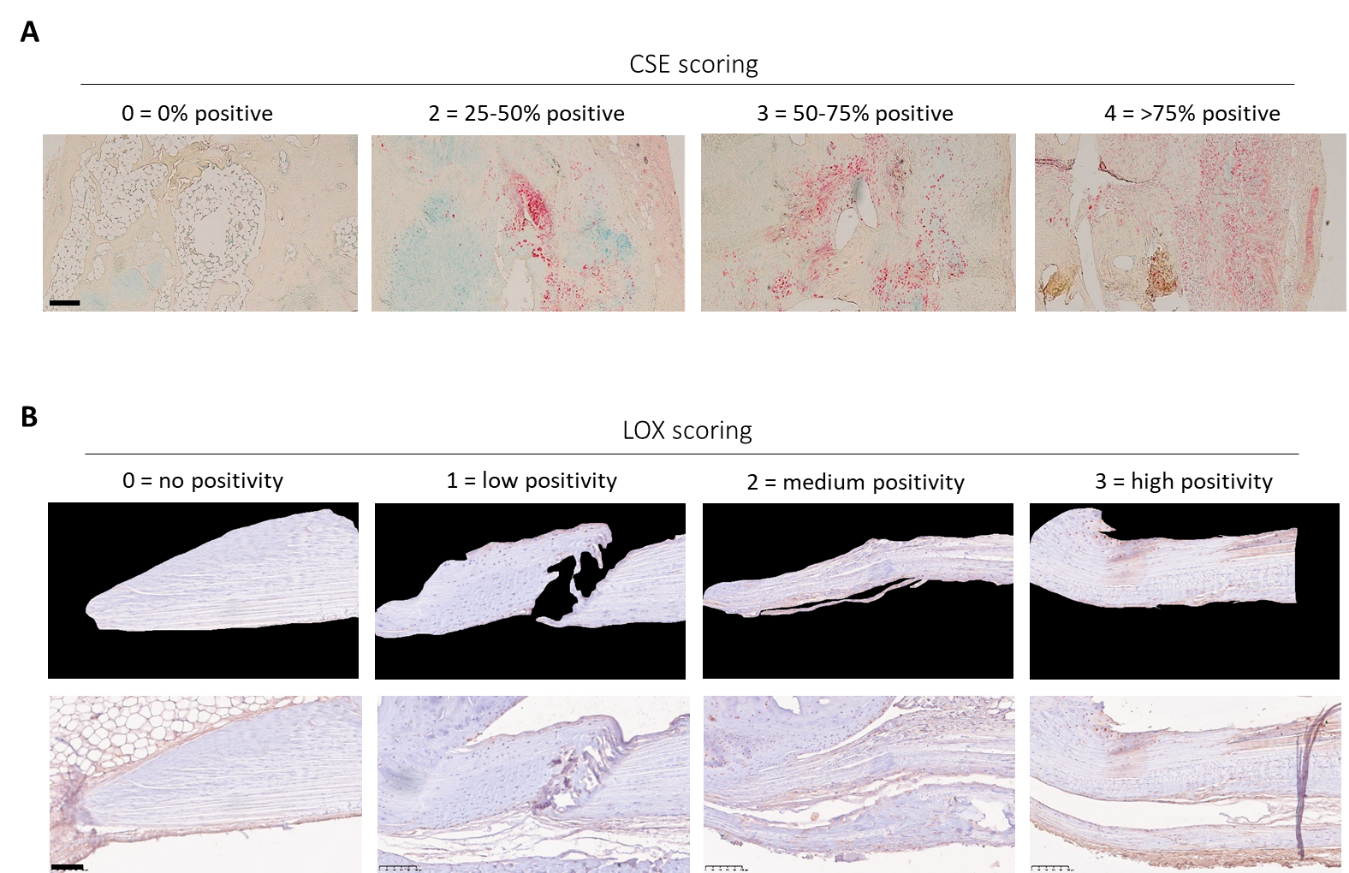


Fig. S3: Scoring system in murine tendons for (A) CSE expression, (B) LOX and LOXL2 expression. Scoring was done on pictures on the top, after masking irrelevant area (black area). Scale bars 100µm.


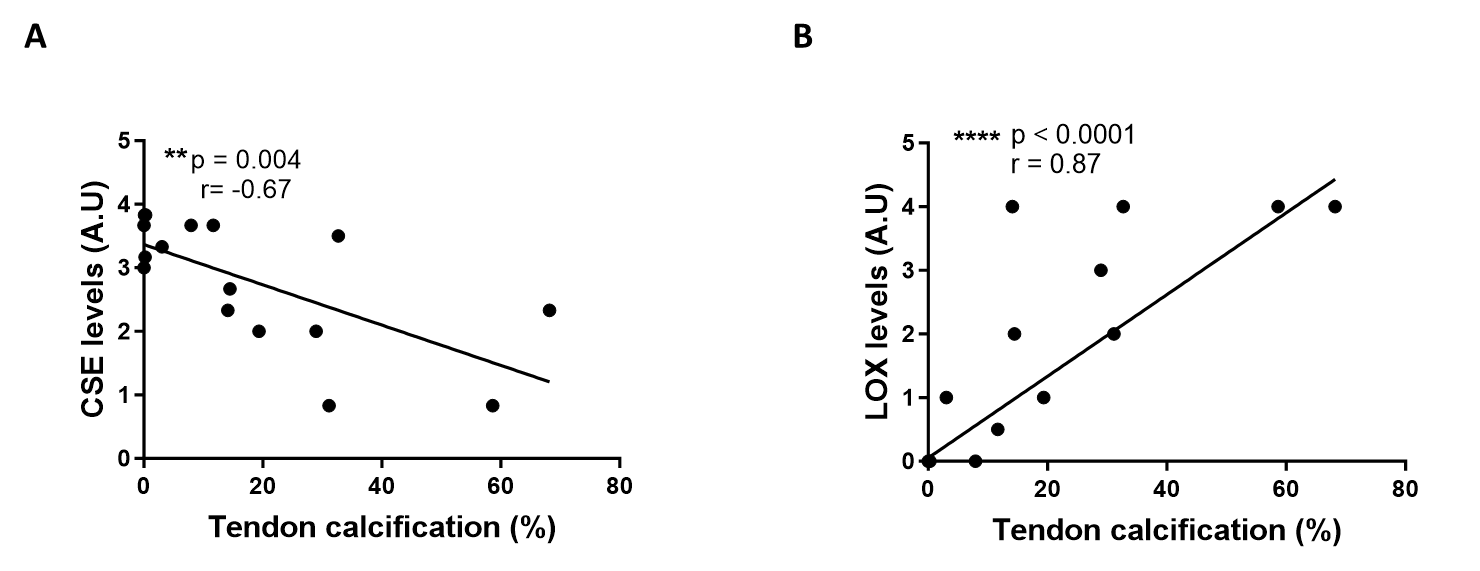


Fig. S4: (A) Correlation graph between CSE (A.U) and tendon calcification (%) in human tendons. Linear regression, n=16. (B) Correlation graphs between LOX expression (A.U) and tendon calcification (%) in human tendons. Linear regression, n=16.


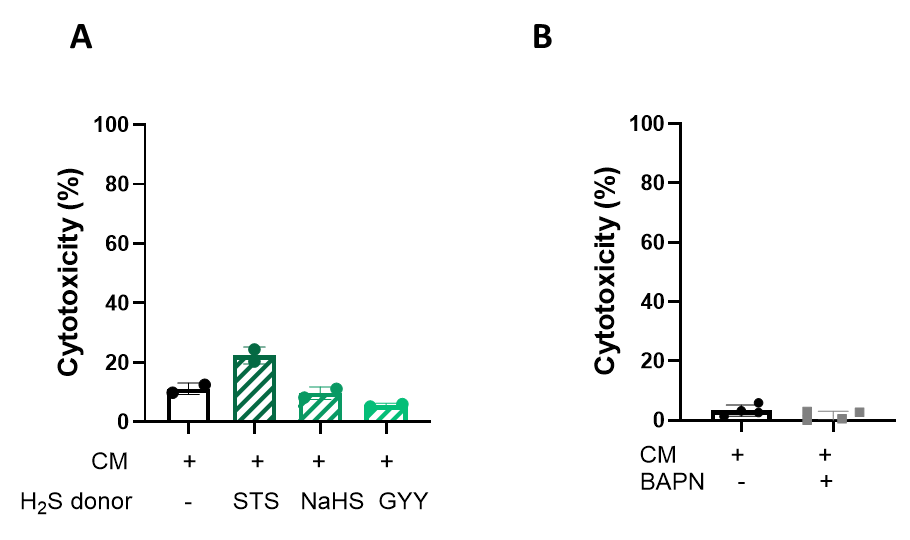


**Fig. S5:** (A) % of cytotoxicity, measured by LDH release, in WT tenocytes cultured for 24 hours in calcification medium and treated or not with H_2_S-donors (STS, NaHS, GYY). n = 2. (B) % of citoxicity, measured by LDH release, in WT tenocytes cultured for 14 days in calcification medium and treated or not with a pan-LOX(L) inhibitor (BAPN). n = 4.
